# Decision Making Cognition in Primary Progressive Aphasia

**DOI:** 10.3233/BEN-2012-0352

**Published:** 2011-12-29

**Authors:** Ezequiel Gleichgerrcht, Teresa Torralva, María Roca, Daniela Szenkman, Agustin Ibanez, Pablo Richly, Mariángeles Pose, Facundo Manes

**Affiliations:** ^1^Institute of Cognitive Neurology (INECO)Buenos AiresArgentina; ^2^Institute of NeurosciencesFavaloro UniversityBuenos AiresArgentina; ^3^Universidad Diego PortalesSantiagoChile; ^4^National Scientific and Technical Research Council (CONICET)Buenos AiresArgentina

## Abstract

We sought to investigate the decision making profile of Primary Progressive Aphasia (PPA) by assessing patients diagnosed with this disease (*n* = 10), patients diagnosed with behavioral variant frontotemporal dementia (bvFTD, *n* = 35), and matched controls (*n* = 14) using the Iowa Gambling Task, a widely used test that mimics real-life decision making. Participants were also evaluated with a complete neuropsychological battery. Patients with PPA were unable to adopt an advantageous strategy on the IGT, which resulted in a flat performance, different to that exhibited by both controls (who showed advantageous decision making) and bvFTD patients (who showed risk-appetitive behavior). The decision making profile of PPA patients was not associated with performance on language tasks and did not differ between sub-variants of the disease (namely, semantic dementia and progressive nonfluent aphasia). Investigating decision making in PPA is crucial both from a theoretical perspective, as it can shed light about the way in which language interacts with other cognitive functions, as well as a clinical standpoint, as it could lead to a more objective detection of impairments of decision making deficits in this condition.

## 1. Introduction

Decision-making is a complex process requiring the interaction of many cortical and subcortical regions. It is widely accepted that the ventral prefrontal cortex (PFC) plays a pivotal role in social and emotional decision-making [1–3]. Additionally, converging evidence indicates the importance of other brain regions, including the dorsal prefrontal cortex and the amygdala, and highlights the relevance of lesion laterality and lesion etiology across regions [4]. The cognitive demands prompted by this complex processes can be difficult to capture with neuropsychological batteries, but in the last decade, the assessment of decision making has prompted the development of new tasks, and it is now a cognitive process that lends itself to laboratory measurement.

We have recently reviewed the way in which decision making is affected in neurodegenerative disease [4]. For instance, patients with Alzheimer disease (AD) seem to engage in random choice-making, leading to non-advantageous outcomes. Basal ganglia degeneration in patients with Parkinson disease (PD) and Huntington disease (HD) deteriorates their efficient decision-making abilities, in part, because the disruption of cortico-subcortical cognitive loops affect their executive performance and their reward-associated learning. In turn, patients with behavioral variant frontotemporal dementia (bvFTD) show a real risk-appetitive behavior characterized by their consistent choice of options presenting immediate reward but long term punishments, which is most likely associated with the degeneration of the prefrontal cortex.

In the current study, we investigated decision making in patients with Primary Progressive Aphasia (PPA). Mesulam originally described PPA as “a slowly pro-

ISSN 0953-4180/12/$27.50 © 2012 – IOS Press and the authors. All rights reserved gressing aphasic disorder without the additional intellectual and behavioral disturbances of dementia. Memory, judgment, and executive function should be intact at least in the two first years of the disease". Although there have been some studies of cognitive performance beyond language functions in PPA [5–7], to the best of our knowledge, however, no research has been conducted on the decision making cognition of patients with this pathology.

PPA is most frequently associated with frontotemporal pathology and three clinical types of aphasia have been described: nonfluent aphasia (PNFA), semantic dementia (SD), and logopenic aphasia. Patients with PNFA exhibit effortful, halting speech characterized by a loss of grammatical complexity and a high frequency of errors. In turn, SD patients show a more fluent speech with a loss semantic knowledge, thus affecting the content of their discourse, which is also reflected in their poor performance on confrontation naming tasks. Because of its relatively recent description, some of the core characteristics of the logopenic subtype are still matter of debate [8,9], but it is generally accepted that these PPA patients present with low speech rates and impaired complex syntactic comprehension and difficulties in naming [10]. The differences between PPA patients are thought to result from distinct patterns of cortical atrophy and their underlying pathological changes. While patients with PNFA tend to show left posterior frontoinsular atrophy [11] and, more typically, FTD-tau pathology [12], SD patients tend to exhibit anterior temporal lobe atrophy [11] and FTLD-TDP pathology [12].

The importance of understanding decision making in PPA is at least twofold. On the one hand, it can further our knowledge about the way in which cortical degenerations leading to prominent language deficits can impact on other higher cognitive functions. Secondly, it bears a fundamental clinical relevance for legal reasons, because as we will argue later in this article, it can contribute to the way we judge faulty decisions made by patients with PPA. For these reasons, the present study sought to investigate the decision making profile of patients diagnosed with PPA and its relationship with language impairment.

## 2. Methods

### 2.1. Participants

Patients with diagnosis of PPA ($n = 10$; 5 PNFA and 5 SD) and bvFTD ($n = 35$), as well as healthy controls ($n = 14$), were consecutively recruited from our clinic. Diagnosis was initially made independently by two experts in the FTD complex (FM and MP) and confirmed in the context of a multidisciplinary meeting (comprising cognitive neurologists, psychiatrists, neuropsychologists and speech pathologists) during which each patient's case is reviewed individually. Inter-reliability diagnosis was excellent (Cohen's kappa = 0.92). BvFTD and PPA diagnosis was made on the basis of published criteria [13] and on the new classification of PPA [10], as one of the authors was involved in said classification. All patients in the former group presented with prominent changes in social behavior and personality as verified by a close relative as well as frontal atrophy on MRI and/or frontal hypoperfusion on SPECT, when available. Classification of PPA patients into PNFA and SD was confirmed by an experienced speech pathologist and linguist expert in neurodegenerative pathologies (DS). Patients included in the present study did not meet criteria for specific psychiatric disorders, as determined by exhaustive psychiatric examination. They were in the mild stages of the disease, as determined by a score of 0.5 or 1 on the Clinical Dementia Severity Rating Scale (CDR) [14]. Healthy controls were matched for age, gender, and years of education, and they reported no history of traumatic brain injury, psychiatric disorders, or substance abuse. All participants gave their informed consent prior to inclusion in this study.

### 2.2. Procedure

The study was initially approved by the ethics committee at the Institute of Cognitive Neurology (Buenos Aires, Argentina) following the ethical standards established by the 1964 Declaration of Helsinki. Participants completed a series of interviews, including neurological and psychiatric assessment, and standard neuropsychological assessment. All participants were administered the Iowa Gambling Task (IGT).

#### 2.2.1. Neuropsychological assessment

Participants in all groups were assessed with an extensive neuropsychological battery assessing (a) general cognitive status, with the Mini Mental State Examination (MMSE) [15], and the Addenbrooke's Cognitive Examination – Revised (ACE-R) [16]; (b) verbal memory through the Rey auditory verbal learning test (RAVLT) [17] and the logical memory subtest of the WAIS-R [18], as well as non-verbal memory with the Rey Complex Figure (RCF) test [17]; (c) attention with the forward digits span task of the WAIS-III [18] and the Trail Making Test Part A (TMT-A) [19]; (4) executive function using the backward digits span test [18], Part B of the Trail Making Test (TMT-B) [19], and the modified version of the Wisconsin Card Sorting Test (WCST) [20]; and (e) language with the Boston Naming Test [21] for naming, the Token Test [22] for comprehension, phonological (letter "P") and semantic (animals) fluency, and the Pyramid and Palm trees test [23] for semantic knowledge.

#### 2.2.2. The Iowa Gambling Task

Bechara et al. [24] developed the Iowa Gambling Task (IGT) in order to provide a more realistic scenario for the assessment of decision-making. Participants are asked to choose between four decks of cards A–D and are instructed that the goal of the task is to maximize their profit with an initial loan of $2000. After picking a card, participants always earn a certain amount of money. However, repeatedly throughout the trails, certain cards may also subtract money (e.g. participant picks a card and is awarded $200 yet loses $50). Participants are unaware that two of the decks are 'advantageous' – cards selected from these decks are associated with either small monetary rewards or, in comparison to the rewards, smaller losses – whereas the other two decks are 'disadvantageous' – cards selected from these decks are associated with either large monetary rewards or even larger losses. As such, an advantageous deck may award the participant with $60 and the deduct $50 (i.e., net gain), while a disadvantageous deck may award $300, but subtract $1200 (i.e., net loss). For this reason, repeatedly selecting cards from the 'advantageous' decks will result in overall profit, whereas repeatedly selecting cards from the 'disadvantageous' decks will result in a net loss over time. Participants are also unaware that they must choose 100 cards before the task is over. For the purposes of performance analysis on the IGT, five blocks of 20 consecutive cards are defined (i.e., cards 1 through 20, cards 21 through 40, and so on), and a net score is calculated for each block as the number of cards chosen from the advantageous decks (C + D) minus the number of cards chosen from the disadvantageous decks (A + B). A total net score is also obtained by adding the individual net scores for each of the five blocks. The IGT is thought to measure decision making beyond the basic executive function capabilities measured by classic laboratory tests. This is supported by the fact that correlations between such executive tasks and IGT performance have not been found consistently throughout the literature, thus suggesting that IGT measures an ability to make decisions that mimics more closely the demands of real-life scenarios (for further discussion on this issue, please see [4]).

### 2.3. Statistical analysis

Demographic and neuropsychological data were compared between the three groups using a one-way ANOVA design followed by Bonferroni post hoc tests when appropriate. When analyzing categorical variables (e.g. gender, recognition), the Freeman-Halton extension of the Fisher exact probability test for 2 $\times$ 3 contingency tables was used. Performance on the IGT was analyzed using a 3 (group) $\times$ 5 (IGT blocks) ANOVA. For comparisons of test performance between subtypes of PPA (SD and PNFA), $U$ Mann-Whitney comparisons were used because of the nonparametric nature of the variables in this sample. The relationship between different variables was calculated using Spearman's correlation coefficient. The $\alpha$ value was set at 0.05, two-tailed for all analyses.

## 3. Results

### 3.1. Demographic and neuropsychological performance

As seen in Table 1, groups were successfully matched for age ($F_{2,54} = 0.72$, $p = 0.49$), gender ($\chi^2 = 0.25$, df = 2, $p = 0.98$) and years of education ($F_{2,54} = 2.23$, $p = 0.17$). Performance on neuropsychological tests was as expected for each patient group. Naming as measured by the BNT ($F_{2,54} = 38.9, p < 0.001$) and semantic fluency differed significantly between PPA and bvFTD patients ($p < 0.001$ and 0.018, respectively). PPA patients also showed a significantly lower performance ($p < 0.01$) on the ACE-R ($F_{2,54} = 17.2$, $p < 0.001$) but not ($p = 0.89$) on the MMSE ($F_{2,54} = 5.15$, $p < 0.01$) since the former test includes a more comprehensive and extensive section on language.

### 3.2. Iowa Gambling Task

As Fig. 1 shows, a 3 (group) $\times$ 5 (IGT blocks) repeated ANOVA design revealed no significant main effect of block ($F_{4,204} = 1.41, p = 0.23$) but both a significant main effect of group ($F_{2,51} = 14.9$, $p < 0.001$) and a significant group $\times$ block interaction ($F_{8,204} = 2.51$, $p = 0.01$). Indeed, a significant difference was found between the groups particularly on block 2 ($F_{2,51} =$

Table 1
Demographic information and neuropsychological test performance. Values are shown as Mean (SD)

| | | | PPA ($n = 10$) | bvFTD ($n = 35$) | CTR ($n = 14$) | Group Comparison | $P$ | CTR vs. PPA | CTR vs. bvFTD | PPA vs. bvFTD |
|---|---|---|---|---|---|---|---|---|---|---|
| Demographics | Age (years) | | 69.6 (8.9) | 68.5 (7.3) | 65.5 (6.5) | $F = 0.72$ | 0.49 | n.s. | n.s. | n.s. |
| | Gender (M: F) | | 5:5 | 18: 17 | 7: 7 | $\chi^2 = 0.25$ | 0.98 | n.s. | n.s. | n.s. |
| | Education (years) | | 16.6 (2.4) | 13.6 (4.5) | 13.9 (3.0) | $F = 2.23$ | 0.17 | n.s. | n.s. | n.s. |
| Cognitive status | ACE-R | | 69.7 (12.6) | 81.9 (10) | 94.5 (5.3) | $F = 17.2$ | $< 0.001$ | $< 0.001$ | 0.001 | $< 0.01$ |
| | MMSE | | 25.9 (2.7) | 26.8 (2.9) | 29.2 (1.0) | $F = 5.15$ | $< 0.01$ | 0.02 | 0.03 | n.s. |
| Memory | Logical Memory | Immediate | 14.3 (11.7) | 16.9 (7.6) | 24.1 (8.3) | $F = 4.39$ | 0.02 | n.s. | 0.03 | n.s. |
| | | Delayed | 9.33 (9.4) | 9.71 (8.6) | 18.9 (7.9) | $F = 5.74$ | $< 0.01$ | n.s. | $< 0.01$ | n.s. |
| | | Recognition | 12.8 (4.0) | 12.9 (4.9) | 16.9 (2.9) | $F = 3.90$ | 0.027 | n.s. | 0.012 | n.s. |
| | RAVLT | Immediate | 22.4 (9.4) | 28.1 (10) | 25.1 (8.9) | $F = 12.3$ | $< 0.001$ | $< 0.001$ | $< 0.001$ | n.s. |
| | | Delayed | 3.63 (3.8) | 3.77 (2.9) | 20.1 (8.9) | $F = 13.2$ | $< 0.001$ | $< 0.01$ | $< 0.001$ | n.s. |
| | | Recognition | 8.13 (4.2) | 9.63 (3.5) | 17.1 (2.9) | $F = 5.51$ | $< 0.01$ | 0.022 | 0.012 | n.s. |
| | RCF | Delayed | 18.2 (8.6) | 6.86 (7.7) | 18.5 (9.1) | $F = 12.9$ | $< 0.001$ | $< 0.01$ | $< 0.001$ | n.s. |
| | | Recognition | 50% hit | 50% hit | 92% hit | $\chi^2 = 0.39$ | 0.04 | 0.04 | 0.04 | n.s. |
| Attention | Digit span forward | | 5.44 (1.6) | 5.94 (1.3) | 5.0 (1.1) | $F = 8.69$ | 0.001 | $< 0.01$ | $< 0.01$ | n.s. |
| | TMT-A (sec) | | 128.4 (181) | 66.4 (31) | 39.4 (16.4) | $F = 3.83$ | 0.028 | n.s. | 0.026 | n.s. |
| Executive functions | Digit span backwards | | 3.33 (0.9) | 3.83 (1.3) | 5.0 (1.1) | $F = 6.61$ | $< 0.01$ | $< 0.01$ | $< 0.01$ | n.s. |
| | Phonologic fluency | | 7.67 (4.1) | 12.4 (7.1) | 17.5 (5.7) | $F = 7.00$ | $< 0.01$ | $< 0.001$ | 0.033 | n.s. |
| | TMT-B (sec) | | 251.1 (162) | 182.8 (72) | 94.1 (44.3) | $F = 8.71$ | 0.001 | $< 0.001$ | 0.012 | n.s. |
| | WCST (total score) | | 3.60 (2.3) | 3.29 (1.8) | 5.6 (0.7) | $F = 7.22$ | $< 0.01$ | n.s. | 0.001 | n.s. |
| Language | BNT (/20) | | 6.43 (7.1) | 17.8 (2.9) | 19.8 (0.4) | $F = 38.9$ | $< 0.001$ | $< 0.001$ | n.s. | $< 0.001$ |
| | Token test (/32) | | 25.8 (5.1) | 22.7 (4.6) | 25.2 (1.1) | $F = 2.82$ | 0.39 | n.s. | n.s. | n.s. |
| | Semantic fluency | | 7.44 (5.3) | 13.5 (6.0) | 20.7 (5.2) | $F = 13.9$ | $< 0.001$ | $< 0.001$ | $< 0.01$ | 0.018 |
| | Pyramid and Palm Trees | | 44.7 (8.6) | 49.6 (3.5) | 51.9 (0.3) | $F = 6.90$ | 0.001 | $< 0.01$ | 0.018 | n.s. |

PPA = Primary Progressive Aphasia; bvFTD = behavioral variant Frontotemporal Dementia; CTR = Control; ACE-R = Addenbrooke's Cognitive Examination – Revised; MMSE = Mini-Mental State Examination; RAVLT = Rey Auditory Verbal Learning Test; RCF = Rey Complex Figure; TMT = Trail Making Test (-A and -B parts); WCST = Wisconsin Card Sorting Test; BNT = Boston Naming Test; n.s. = not significant.

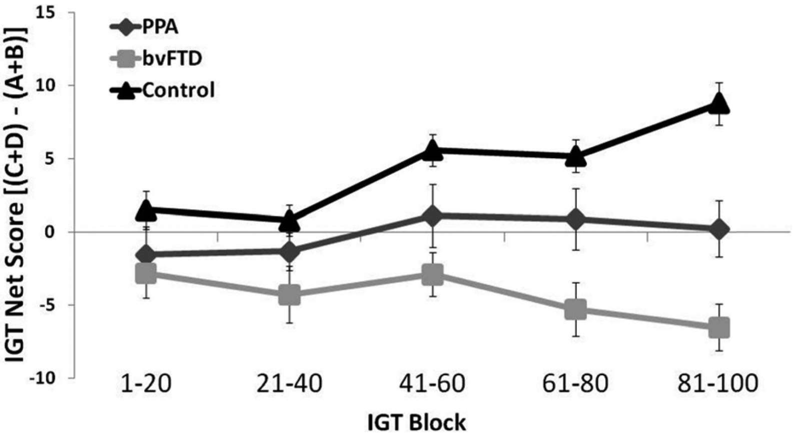

Fig. 1. *Mean* (*SEM*) group performance on the IGT, as measured by the net score ("good decks" minus "bad decks") on each 20-card block.

4.05, $p = 0.023$), block 3 ($F_{2,51} = 5.31$, $p < 0.01$), block 4 ($F_{2,51} = 8.14$, $p = 0.001$), and block 5 ($F_{2,51} = 15.3$, $p < 0.001$). In all of these cases, bvFTD patients were outperformed by controls (block 2: $p = 0.021$; blocks 3 and 4: $p < 0.01$; block 5: $p < 0.001$). Controls showed significant differences from PPA patients exclusively on block 5 ($p = 0.027$). PPA patients showed a significantly better performance than bvFTD patients particularly on blocks 4 ($p = 0.05$) and block 5 ($p = 0.024$).

Total net score on the IGT (i.e., the sum of the five block net scores) significantly differed between the groups ($F_{2,51} = 15.35$, $p < 0.001$), with PPA patients differing significantly from bvFTD patients ($p = 0.036$) but not from controls ($p = 0.076$), although the bvFTD and control groups differed significantly ($p < 0.001$)

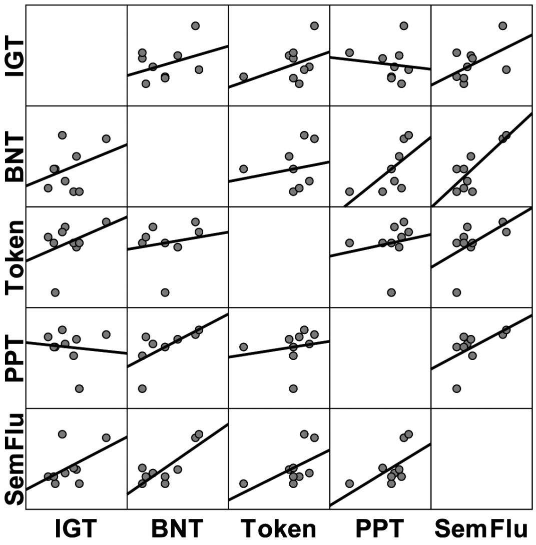

Fig. 2. Correlation matrix for language variables and total net score on the IGT (Iowa Gambling Task). No significant correlations were found. BNT = Boston Naming Test; Token = Token Test; PPT = Pyramid and Palm Trees; SemFlu = Semantic Fluency.

between each other.

### 3.3. *Correlation analyses* (*Fig. 2*)

No significant correlations were found within PPA patients between the total net score on the IGT and the BNT ($r = 0.08, p = 0.83$), Token test ($r = 0.16, p = 0.65$), PPT ($r = -0.04, p = 0.91$), or semantic fluency ($r = 0.36, p = 0.31$). Yet, when looking at correlations between language tasks, performance on the PPT was significantly associated with BNT scores ($r = 0.74$, $p = 0.015$) and the number of words produced on the semantic fluency task ($r = 0.64$, $p = 0.04$). Correlations sought between other scores derived from the IGT (e.g. block 5 score) and language tasks were also non-significant.

### 3.4. *PNFA vs. SD*

No significant differences were found between PNFA and SD patients on the IGT for any of the blocks (1: $U = 6.0, p = 0.26$; 2: $U = 7.0, p = 0.35$; 3: $U = 9.5$, $p = 0.61$; 4: $U = 11.0$, $p = 0.91$; and 5: $U = 10.5$, $p = 0.76$). The groups did not differ on the total IGT net score either ($U = 11.0$, $p = 0.91$). Correlations between IGT performance and language variables remained non-significant within each PPA subgroup analyzed independently.

### 3.5. *Positive vs. negative total net score*

In order to further explore the possibility of differential decision making profiles within the PPA population, patients were divided into two groups based on whether they obtained a positive (i.e., greater than zero; $n = 4$) or negative (i.e., lower than zero; $n = 6$) total net score on the IGT. No significant differences were found on any of the demographic variables (age: $U = 6.0, p = 0.67$, gender: $\chi^2 = 0.74, p = 0.39$, education: $U = 7.5$, $p = 0.71$, and age at onset: $U = 6.50$, $p = 0.26$). Similarly, positive and negative IGT net score PPA patients had comparable cognitive status (ACE-R: $U = 10.0, p = 0.76$, MMSE: $U = 9.0, p = 0.91$). No significant differences were found on the BNT ($U = 4.0$, $p = 0.63$), the Token test ($U = 3.0$, $p = 0.51$), semantic fluency ($U = 7.50$, $p = 0.71$), and the PPT ($U = 3.0, p = 0.51$). Similarly, the groups had comparable performance on tasks of memory, attention, and executive functions (all $p > 0.24$).

## 4. Discussion

Decision making cognition, as assessed using the Iowa Gambling Test, was impaired in patients with PPA. In the present study, patients with PPA showed a decision making profile characterized by lack of adoption of an advantageous strategy, which led to a "flat performance" on the Iowa Gambling Task. Said flat performance is characterized by a net score (i.e. advantageous – disadvantageous choices) that is close to null throughout the task, reflecting the fact that card choices favored neither advantageous nor disadvantageous decks. This profile differed significantly from that of healthy control subjects, who showed more frequent choice-making from the "good" decks, resulting in an overall gain. Importantly, the decision making profile of PPA patients also differed significantly from that of patients with bvFTD, since the latter group showed risk-appetitive behaviors by which long term losses were masked by immediate rewards, a decision making pattern which has been consistently reported in the literature for this population [25,26]. It must be noted that because of this proximity to a "zero" net score, PPA patients could be perceived as being more risk-appetitive than controls. In this sense, it is important to consider that PPA patients showed a significantly less risky decision making profile than the bvFTD group, a truly risk-appetitive population. This suggests that risk taking among PPA patients is most likely unrelated to an inability to foresee the negative outcome of risky choices, as it occurs in bvFTD.

PPA performance on the IGT seemed neither to be related to their language deficits nor to differ between subtypes of PPA. Several hypotheses could potentially explain the flat IGT performance exhibited by PPA patients in our study. First, it could be the case that language deficits affected patients' understanding of the task. This seems unlikely, however, as no significant differences were found between the groups on the Token test, which is widely used to measure comprehension of verbal instructions. Moreover, while one would expect a positive significant correlation between IGT scores and the Token test (i.e., the worse comprehension is, the poorer the performance on decision making), no such correlation was found. Second, deficits on other language functions other than comprehension could be influencing performance on the IGT directly. But again, no significant correlations were found between the IGT and other language variables, including semantic fluency, confrontation naming, and semantic knowledge. Furthermore, when correlation analyses were conducted for each variant (i.e. SD and PNFA) independently, correlations remained non-significant. As well, when PPA patients were classified based on whether they had an overall positive or negative net score on the IGT, no significant differences emerged on any of the language variables. Thus, given that performance on the IGT appears to be unrelated to language abilities, it is likely that the non-advantageous, non-risky (i.e. "flat") decision making profile of PPA patients is not the *direct* result of language deficits typical of this condition.

Perhaps a more promising explanation, given the complex neural networks underlying the decision making process, is that language deficits may have an *indirect* influence on IGT performance. For instance, Rogalski et al. [27] have shown that aphasia in PPA patients contributes to the difficulty they experience in choosing items within the same semantic class. One can thus hypothesize that PPA patients may have been unable to distinguish the potential differences between several decks of cards; perhaps, these were all interpreted as part of the same semantic category (i.e., they were unable to process the differences between the "safe" and the "risky" decks on the IGT). Furthermore, it has been recently reported that PPA patients have difficulties in the representation of quantities in real-life scenarios [28], which could have also contributed to the decision making profile found in this study. On top of the direct and indirect influences of language deficits on decision making, it is also plausible that a shared underlying mechanism affects both decision making and language simultaneously. After all, there are some major common features between both cognitive processes. One such feature, for instance, is the need to access mental representations, which are essential both for language content and for the evaluation of distinct choices (in this case, advantageous vs. disadvantageous) when making a decision. If shared mechanisms are indeed affected in PPA patients, then language and decision making, as well as any other processes feeding on such mechanisms, are prone to being impaired.

These preliminary reflections on the influence of language impairment on IGT performance must of course be interpreted with caution given the small sample size of PPA patients in this study. Of special interest is the prediction that semantic deficits may diminish performance by means of an inability to properly and differentially categorize "safe" vs. "bad" decks, which must be tested in future studies using a larger PPA population of SD patients, whose performance – but not that of PNFA patients – should be affected by impairments in their semantic ability.

The potential role of cognitive deficits typical of PPA patients in decision making performance need not be limited to language problems alone. For example, Banks and Weintraub [29] found a loss of self-awareness in PPA patients, which could also affect performance on the IGT, especially if patients feel their cognitive assessment is useless or a waste of time. In fact, the "flat" profile exhibited by PPA patients in this study resembles that of patients with psychiatric disorders - such as euthymic bipolar patients – who tend to choose randomly between good and bad decks [30, 31]. In this regard, future studies should attempt to compare the performance on the IGT of patients with PPA and with psychiatric disorders in order to determine whether there is a potential common denominator between these conditions that could explain such flat performance. Naturally, it is likely that the performance exhibited by PPA patients on the IGT is the combined result of multiple factors rather than one in particular, including those detailed above, as well as other underlying mechanisms that are yet to be identified.

The findings of the present study must be considered in the context of certain caveats, of which the small sample size of our PPA group is a major limitation. However, this is the first empirical study to examine decision making cognition in PPA. The results hereby presented are supported by the fact that the groups were accurately paired with healthy controls, as well as the fact that we did find robust significant effects in terms of performance on the IGT. Importantly, the groups in this study are similar in size to those employed in the other relevant reports on the neuropsychological aspects of PPA (ten patients in ref. [7], five patients in ref. [32]; etc.). Future studies should try to replicate these findings in larger populations, paying particular attention to potential differences between subtypes of PPA. While the diagnosis of patients with PPA in our study was based solely on clinical assessment and not on pathological analyses, it is important to note that patients were longitudinally studied as part of our FTD program, and all of them went on to exhibit the typical clinical syndrome of PPA.

In this seminal study on the decision making profile of patients with PPA, we have found a very distinct performance pattern compared to that exhibited by healthy controls and to patients with another variant under the umbrella of frontotemporal dementias. Identifying the way in which patients with this condition make decisions in real life has relevant implications from many angles. Theoretically, it provides information about the potential relationship between patterns of cortical degeneration, language deficit profiles, and impairments in decision making. By the same token, it can provide evidence for a possible dissociation between performance on language and decision making tasks, which can further help us dissect the complexity of the frontal and temporal circuitry involved in these higher cognitive processes. From a clinical perspective, our findings stress the need to include measures such as the IGT as part of neuropsychological assessment batteries for PPA patients. Among other measures aimed at detecting executive and social cognition deficits [33], the IGT has been shown especially capable of detecting cognitive and behavioral impairments otherwise missed by classical laboratory tests [34]. Understanding the decision making profile of PPA patients is also fundamental in the development of evidence-based policies regarding behavioral problems in this population. If patients affected by this condition fail to make advantageous choices, to what extent can they be considered responsible for their actions? Can they be found guilty for committing a crime? Should the medical decisions considering their own health be left to the patients' own criteria? How should we proceed should the relatives of a patient argue that he/she can no longer take care of the family's financial matters? The answers to these and other similar important questions are not without controversy, but by furthering our understanding of decision making in patients with PPA, we will be able to provide more solid and consistent arguments to contribute towards policy making that will eventually affect the everyday living of these patients and their beloved ones.

## Acknowledgements

The present study was supported by a FINECO grant.
